# Supplementary material for: Loss of TOP3B leads to increased R-loop formation and genome instability
Source: Open Biol. 2019 Dec 4;9(12):190222. doi: 10.1098/rsob.190222 (PMC6936252; doi:10.1098/rsob.190222)
Supplement: Figure S1 [file rsob190222supp1.pdf]

Figure S1

(a)

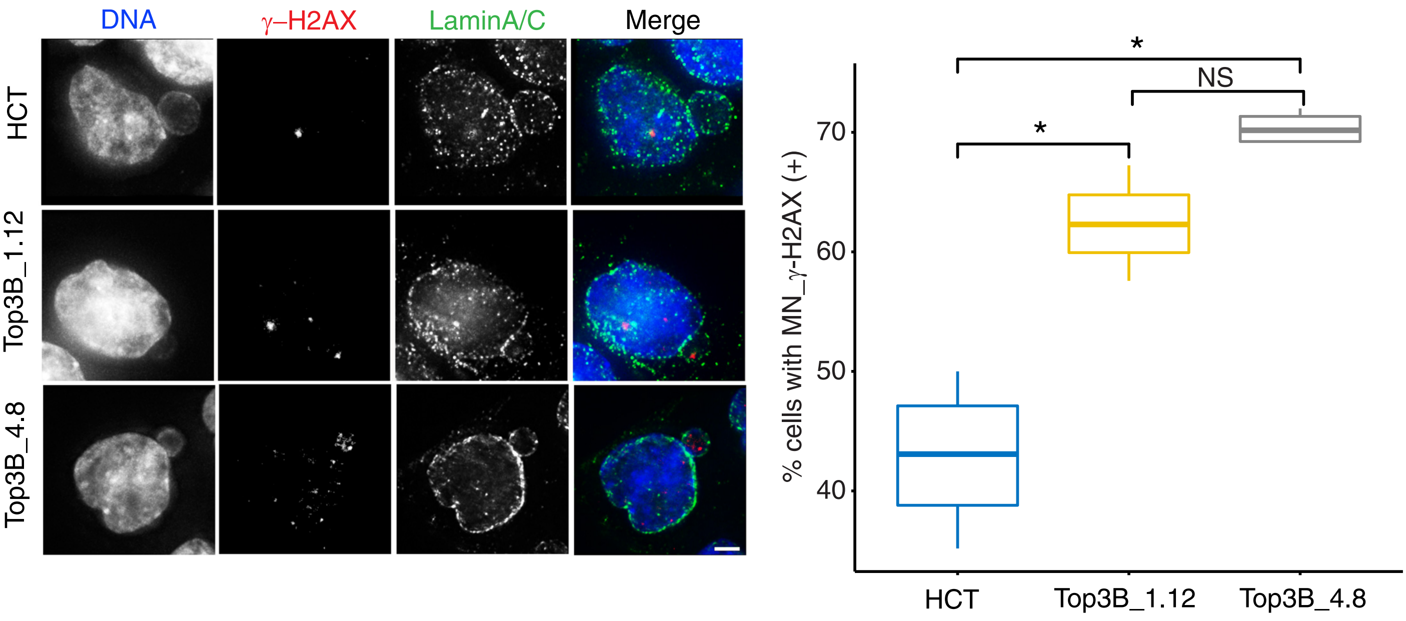

(b)

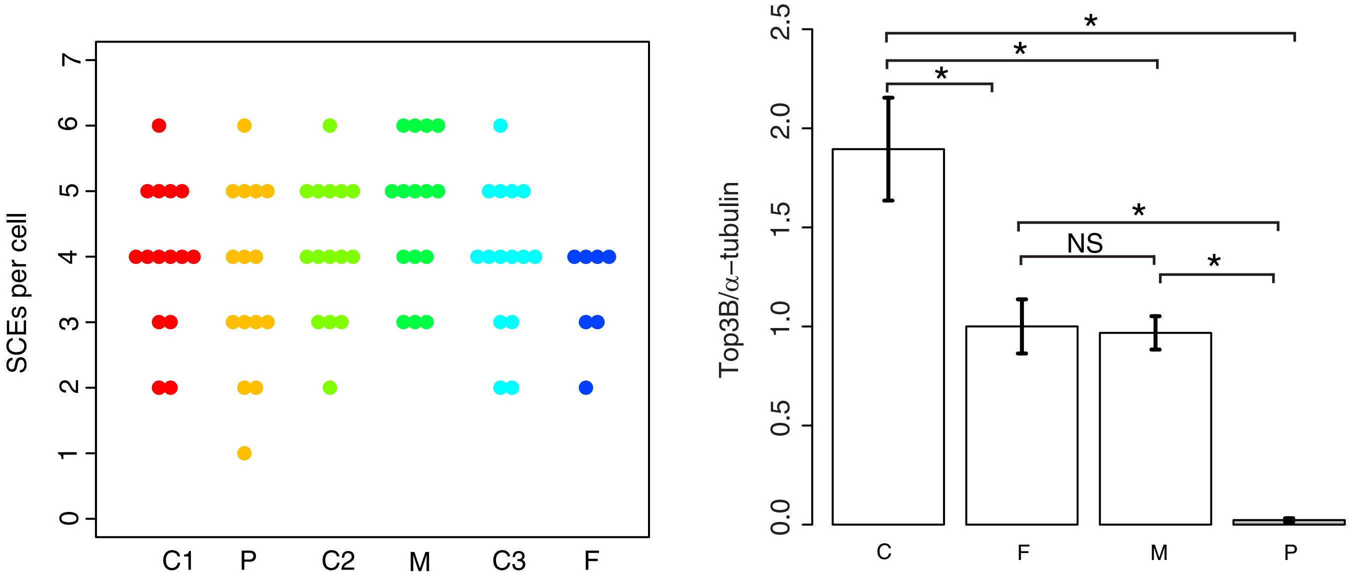

(c)

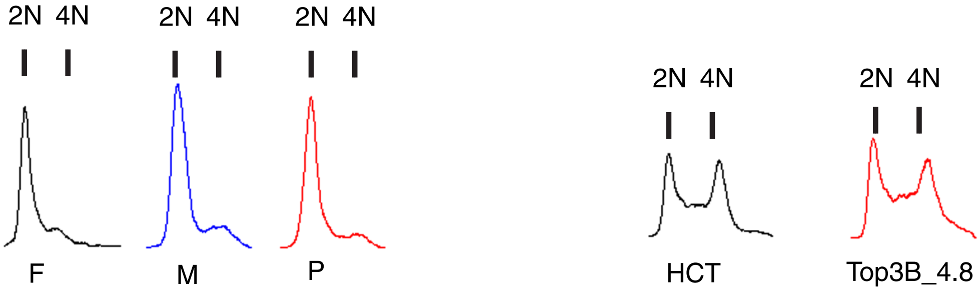

Figure S1. (*a*) Representative images of HCT116 control (HCT) and HCT116 TOP3B null clones stained for lamin A/C (green), γ-H2AX (red) and DNA (DAPI, blue). Right, cells with micronuclei displaying γ-H2AX (MN_γ-H2AX) were scored from three independent experiments with at least 150 cells from each experiment. * denotes $P<0.05$. NS: no significant difference. Scale bar equals 5 μm. (*b*) Left, sister chromatid exchanges were counted from 15 cells individually from age-matched-controls (C1, C2 and C3), parental controls (F, M) and patient (P) samples. Right, intensity measurement of TOP3B, with α-tubulin as the loading control from three different immunoblots of control (C), parental heterozygote controls (F=father, M=mother) and homozygous $TOP3B^{-/-}$ patient (P) lymphoblasts as shown in Fig. 1B. (*c*) FACS analysis shows no difference in ploidy between TOP3B null lymphoblast and HCT116 TOP3B null cells versus controls (F, M: $TOP3B^{+/-}$, P: $TOP3B^{-/-}$ and wild-type HCT116 $TOP3B^{+/+}$ (HCT), HCT116 $TOP3B^{-/-}$ (Top3B_4.8)).#
