# Supplementary material for: Loss of TOP3B leads to increased R-loop formation and genome instability
Source: Open Biol. 2019 Dec 4;9(12):190222. doi: 10.1098/rsob.190222 (PMC6936252; doi:10.1098/rsob.190222)
Supplement: Figure S2 [file rsob190222supp2.pdf]

Figure S2

(a)

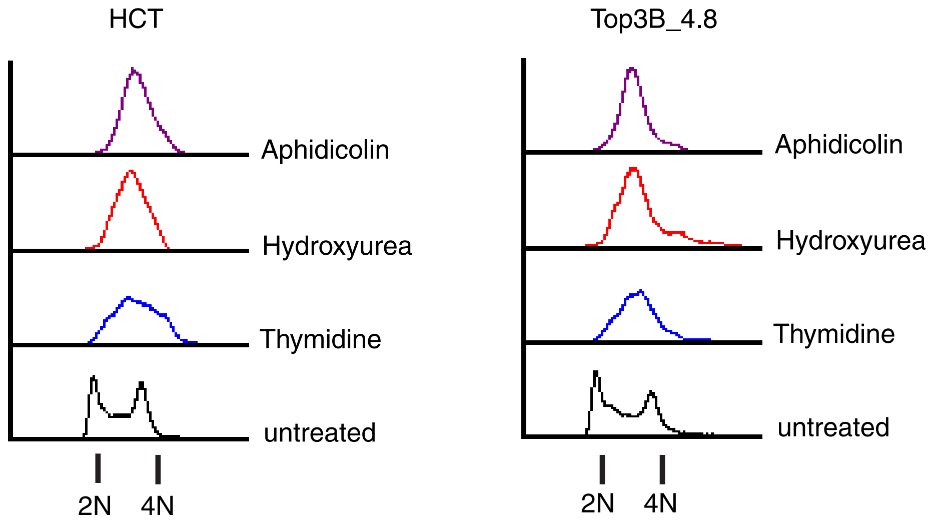

(b)

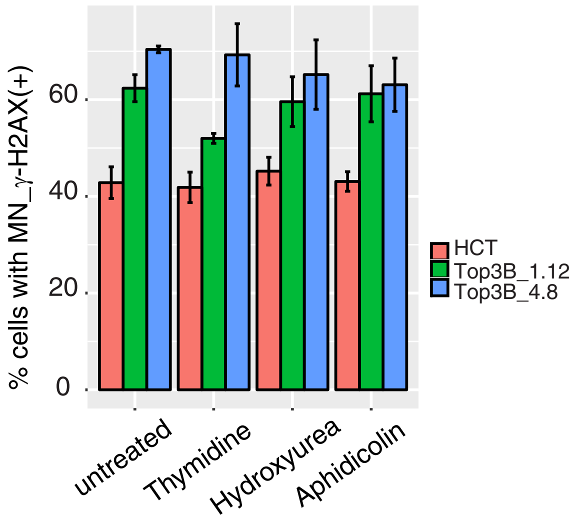

(c)

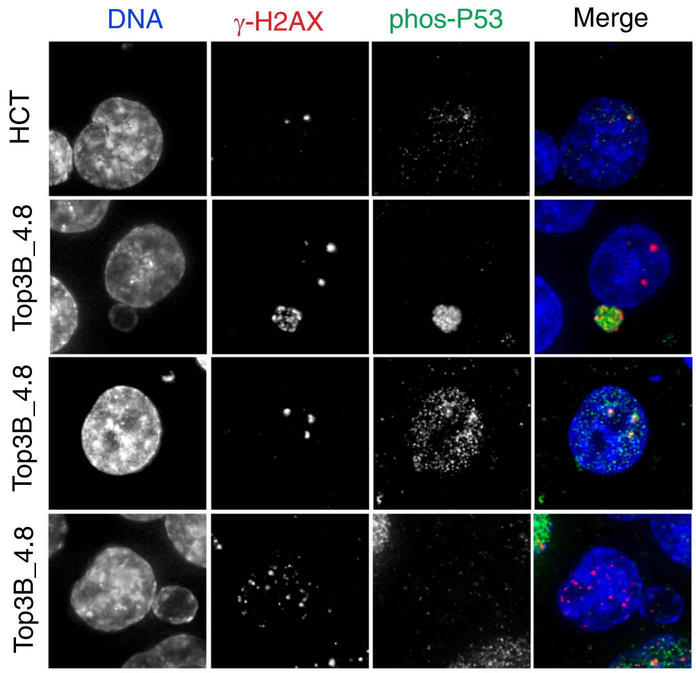

Figure S2. (*a*) FACS analyses of HCT116 control (HCT) and TOP3B null clone (Top3B_4.8) treated with aphidicolin, hydroxyurea, thymidine, or no drug (untreated). (*b*) Quantification of cells with micronuclei displaying#γ-H2AX#(MN_γ-H2AX) in HCT116 control (HCT) and TOP3B null cells (Top3B_1.12 and Top3B_4.8) after recovery from thymidine, hydroxyurea or aphidicolin treatment. Data are from three independent experiments, with at least 150 cells scored from each experiment. (*c*) Representative images of HCT116 control (HCT) and TOP3B null clones (Top3B_4.8) stained for γ-H2AX (red) and phospho-P53 (green).
